# Application of Virtual Reality to Home-Visit Rehabilitation for Patients With Chronic Musculoskeletal Pain: A Single-Group Pre-post Comparison Study

**DOI:** 10.7759/cureus.80386

**Published:** 2025-03-11

**Authors:** Hiroki Funao, Ryo Momosaki, Mayumi Tsujikawa, Eiji Kawamoto, Ryo Esumi, Motomu Shimaoka

**Affiliations:** 1 Department of Molecular Pathobiology and Cell Adhesion Biology, Mie University Graduate School of Medicine, Tsu, JPN; 2 Department of Practical Nursing, Mie University Graduate School of Medicine, Tsu, JPN; 3 Department of Rehabilitation Medicine, Mie University Graduate School of Medicine, Tsu, JPN; 4 Department of Nursing, Suzuka University of Medical Science, Suzuka, JPN; 5 Department of Intensive Care Medicine, Mie University Hospital, Tsu, JPN; 6 Department of Emergency Medicine, National Hospital Organization Mie Chuo Medical Center, Tsu, JPN

**Keywords:** chronic musculoskeletal pain, feasibility study, homebound patient, home-visit rehabilitation, virtual reality

## Abstract

Objective

Virtual reality (VR) is increasingly used to alleviate pain during the rehabilitation of patients with chronic musculoskeletal pain. Previous studies on the application of VR to rehabilitation have reported improvements in pain, functional impairment, and psychological status of patients. However, the focus of many previous studies was on short-term effects and rehabilitation in hospitals. Studies that report home-based rehabilitation for mid- to long-term periods are lacking. Hence, the aim of the study was to investigate the feasibility and safety of applying VR for the home-visit rehabilitation of these patients.

Methods

A single-group pre-post comparative study was conducted at two home healthcare agencies in Japan. Six female participants (mean age: 76.5 years) with chronic musculoskeletal pain underwent 10 sessions of VR-applied home rehabilitation over 10 weeks. In the intervention, a standalone VR headset (MetaQuest 2^TM^; Meta Platforms Inc., Menlo Park, CA, USA) was used to view natural landscape content during rehabilitation. Pain levels, heart rate variability (HRV), motivation for rehabilitation, mood states, Pain Catastrophizing Scale (PCS) scores, and quality of life (QoL) were measured at various time points before, during, and after the VR interventions. All variables were summarized as means and standard deviations, medians and interquartile ranges, or frequencies and percentages, as appropriate.

Results

All participants completed the rehabilitation sessions without dropping out or experiencing adverse effects, thereby supporting the feasibility and safety of the intervention. Pain levels during rehabilitation significantly decreased compared with baseline levels, showing reductions of more than 4.5 points on the Numerical Rating Scale (NRS; 0-10). The HRV values showed inconsistent trends: an increase and a decrease in the parasympathetic and sympathetic nerve indices, respectively, between the baseline and the first intervention point, revealing a shift towards parasympathetic dominance, whereas no clear trend was observed from the 2nd to the 10th interventions. The motivation for rehabilitation in all patients remained strong, and intrinsic regulation was the dominant factor. The mood states of all patients remained stable within the healthy range throughout the study period. PCS scores initially increased; however, they decreased with time. Regarding QoL, mental health scores remained high, whereas physical and social functioning declined and improved, respectively.

Conclusions

The VR-applied home-visit rehabilitation is a feasible and safe approach for patients with chronic musculoskeletal pain. The intervention may reduce pain during rehabilitation; however, its effects did not persist long enough to improve constant baseline pain or other psychological factors. Further studies with larger sample sizes and appropriate control groups are required to confirm the effectiveness and long-term benefits of this approach.

## Introduction

Chronic musculoskeletal pain is defined as pain that results from a disease process that directly affects the bones, joints, muscles, or related soft tissues and persists or recurs for more than three months [1]. Rehabilitation has been shown to be effective in improving chronic musculoskeletal pain [2-4]; however, the increase in exercise-induced pain and fear of exercise due to pain are challenges that hinder effective rehabilitation [5]. Furthermore, as indicated by the fear-avoidance model of pain [6], chronic pain resulting from physical disabilities and diseases, as well as exercise-induced pain associated with rehabilitation, can increase anxiety and fear in patients. Thus, they avoid physical activities, including rehabilitation. The resulting inactivity and depression eventually exacerbate the chronic pain.

Recently, virtual reality (VR) has been applied in the rehabilitation of chronic musculoskeletal pain. VR-assisted rehabilitation has been reported to have physical effects, such as reducing pain and improving functional disorders, as well as psychological effects, including reducing exercise phobias, compared with normal rehabilitation [7,8]. However, only the short-term effects have been studied in many previous reports; hence, the medium- to long-term effects are yet to be fully elucidated. Furthermore, many previous studies were performed in hospitals, medical institutions, and rehabilitation facilities, with only a limited number of studies conducted in home rehabilitation settings.

Many patients who are homebound have chronic musculoskeletal pain and functional disorders. Furthermore, social participation is often limited, leading to anxiety and depression [9,10]. These physical and psychological conditions make it difficult to alleviate chronic pain [11]. In addition, patient homes are not ideal for effective rehabilitation programs, unlike hospitals. Consequently, patients find it difficult to engage in rehabilitation and are more likely to lose their motivation [12]. Maintaining patient motivation becomes more difficult when the home-based rehabilitation sessions are prolonged [13]. It is important to alleviate pathological psychosocial factors, such as depression, anxiety, and reduced motivation, as well as relieve pain during treatment for patients with chronic musculoskeletal pain who require rehabilitation at home due to difficulties in visiting a hospital because of illness or disability [14].

Therefore, the aim of this study was to investigate the feasibility and safety of applying VR in home-visit rehabilitation for patients with chronic musculoskeletal pain who are homebound. Applying VR in this setting may help promote rehabilitation, thereby effectively improving chronic musculoskeletal pain. The results of this study may provide insights into the medium- to long-term use of VR in home-based rehabilitation settings.

## Materials and methods

Study design

This single-group pre-post comparison study was conducted to evaluate the feasibility of the potential application of VR in the home-visit rehabilitation of patients with chronic musculoskeletal pain. The study was conducted in line with the research protocol previously reported by the authors [15]. It was approved by the Clinical Research Ethics Review Committee of Mie University Hospital, Tsu, Japan (approval no. H2021-143) and registered as a clinical trial with the University Hospital Medical Information Network (UMIN; UMIN000045039). All participants were informed of the details of the study and provided face-to-face informed consent before the study began.

Setting

This multicenter study was conducted at two home healthcare agencies that provide home-visit rehabilitation services in Ise and Yokkaichi, Mie Prefecture, Japan. Data were collected from participants between April 14, 2022, and October 4, 2023.

Participants

Inclusion Criteria

Patients who met all the following criteria were enrolled in the study: (1) musculoskeletal pain that persisted or recurred for more than three months; (2) received home-visit rehabilitation for three months or more; (3) adequate visual and auditory functions to use the VR equipment; (4) adequate cognitive and perceptive functions to answer questions measuring subjective symptoms; (5) 20 years of age or older at the time of consent; and (6) provided free and voluntary written consent to participate in the study after being fully informed.

Exclusion Criteria

Patients with a history of adverse events due to the use of VR devices were excluded.

Recruitment Process

Participants were recruited from two home healthcare agencies. The representatives of the home healthcare agencies consecutively selected patients who met the criteria. The researchers visited the patients and screened those who provided consent to participate in the study. Those who met the criteria were enrolled as research participants. Notably, the study period coincided with the coronavirus disease (COVID-19) pandemic. Therefore, the researchers explained this to the participants and took the following actions: standard infection control measures were observed during the researchers' visit, research equipment was controlled and disinfected by the researchers, and the possibility of COVID-19 infection in relevant personnel was considered. If there was a concern that a person might have been infected with COVID-19, the visit was canceled or postponed.

Sample Size

In this study, considering previous studies where VR was used to relieve pain [16-19] and as an intervention for rehabilitation [20,21], a sample size of 40 cases was set, assuming a dropout rate of 15%.

Interventions

The intervention used in this study was the VR-applied home-visit rehabilitation. The VR intervention was applied to the participants during the same rehabilitation procedures that they usually underwent, including range of motion exercises and stretches. A typical rehabilitation session lasted approximately 40-60 minutes. All sessions consisted of the first and second parts. At the beginning of the first part, physical assessments of the participants' blood pressure and symptoms were carried out. These assessments took approximately 5-10 minutes. After the assessments, range of motion exercises and stretches were performed, taking approximately 10-20 minutes. In the second part of the session, walking training and activities of daily living (ADL) training were carried out according to the degree of the participants' physical activities. The training lasted for approximately 20-30 minutes. The VR intervention was applied to the participants for as long as possible during the session.

VR Device

**Figure 1:**
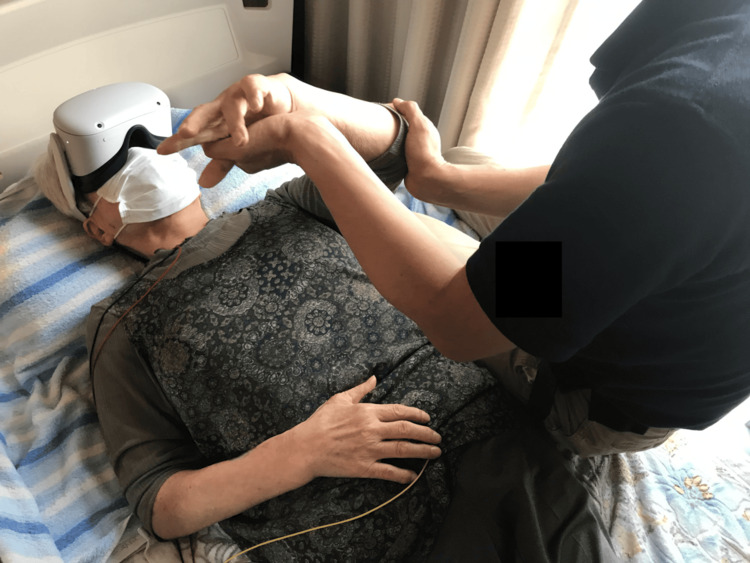
A participant wearing a VR device Image showing a participant wearing the MetaQuest 2^TM^ during the home-visit rehabilitation procedure that she usually receives. Image credit: This figure is an original creation by the author, Hiroki Funao. The image was created using photography. VR, Virtual reality

The VR device used in this study was a MetaQuest 2^TM^ (Meta Platforms Inc., Menlo Park, CA, USA), a standalone wireless device that does not need to be connected to an external computer and is powered by internal batteries. The participants wore the MetaQuest 2^TM^ during their normal rehabilitation procedures (Figure 1). The VR device was managed and brought to the participants' homes by the researcher. If any of the participants had difficulty setting up and fitting the VR device, the researcher assisted them.

VR Contents

**Figure 2:**
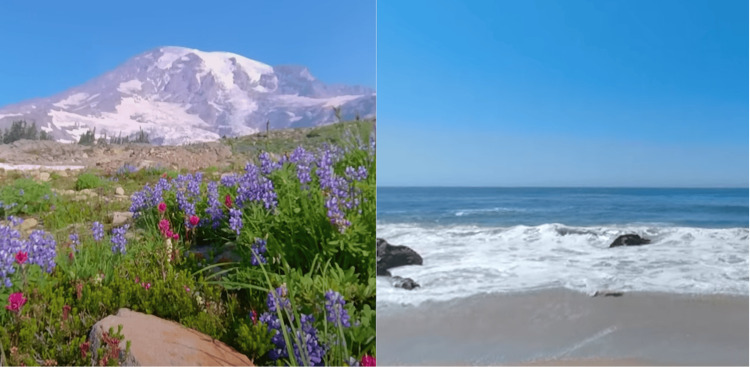
Typical VR contents viewed with the VR device These images show typical VR contents that the participants watched with the MetaQuest 2^TM^ in this study, which included landscapes such as flower fields, beaches, forests, and plateaus (Youtube VR^TM^) [22]. Image credit: This figure is an original creation by the author, Hiroki Funao. The images were created by capturing an application screen using the screenshot function on the MetaQuest 2^TM^. VR, Virtual reality

The content used in this study was selected from natural landscapes available on YouTube VR (https://vr.youtube.com) (Figure 2) [22], because the VR content of natural landscapes has positive psychological effects that promote pain relief [23,24]. In addition, the content was selected based on the following criteria: (1) it does not change the viewpoint frequently or violently and avoids viewing for more than 20 minutes to prevent adverse effects, such as VR sickness [25,26]; (2) it does not require interactive operations during VR viewing to avoid interference with the rehabilitation procedures; and (3) it allows participants to watch in a supine or seated position to avoid the risk of falling or other accidents, as wearing the MetaQuest 2^TM^ blocks their full field of vision.

The researcher observed the participants to evaluate whether there were any problems with using the VR before the VR content began. If any adverse effects occurred, VR home-visit rehabilitation was immediately stopped, and medical attention was provided if necessary. The study was also terminated if any participant developed adverse effects at any stage before completing the entire intervention, and that participant was excluded.

Data collection

At baseline, data were collected during regular home-visit rehabilitation (without VR). Following the baseline measurement, VR-applied home-visit rehabilitation was performed weekly, 10 times in total. Participant characteristics, VR implementation metrics, pain scores, heart rate variability (HRV), motivation levels for rehabilitation, mood states, catastrophic thoughts about pain, and quality of life (QOL) were obtained during the data collection. The researcher observed the participants during home-visit rehabilitation and collected data. After completing the 10 sessions, the participants underwent the usual home-visit rehabilitation, and no follow-up was conducted, except in cases of adverse events.

Participant Characteristics

Participant characteristics included age, sex, ADL, disease as the main cause of pain, and site of pain. The Barthel Index (BI) was used to evaluate ADL and was categorized as follows: 100 = independent, 75-100 = mildly dependent, and 0-70 = moderately or severely dependent [27]. The index was used to assess an individual's ability to perform 10 basic tasks. Data were collected during the baseline period.

VR Implementation Metrics

The duration of the VR interventions and dropout rates were measured. Subjective adverse effects included pressure pain at the site where the VR device is worn, muscle strain from the neck to the shoulders caused by the VR device load, and VR sickness [25]. To measure the physical burden of wearing a head-mounted display, subjective adverse effects were assessed using the Numerical Rating Scale (NRS), as previously shown in patients at home [28]. Data were collected for each VR intervention.

Degree of Pain in Home-Visit Rehabilitation

Pain was assessed using an NRS ranging from 0 to 10, in which 0 indicates no pain at all, and 10 indicates the most intense pain experienced to date [29]. Pain was measured at three time points per rehabilitation session (before, during, and after rehabilitation). For every home rehabilitation visit, the baseline values for each pain type were calculated from the medians of the three home-visit rehabilitation sessions before VR-assisted rehabilitation began.

Heart Rate Variability (HRV)

HRV represents the ability of the heart to respond to physiological and environmental stimuli and is a psychophysiological measurement of reactivity to stress [24]. The parameters measured were heart rate (HR), sympathetic nerve index (low-frequency (LF)/high-frequency (HF) ratio), and parasympathetic nerve index (HF). Electrocardiogram (ECG) recordings of these parameters were performed at baseline and during each VR intervention. The baseline values were calculated from the means of the three home-visit rehabilitation sessions before VR-assisted rehabilitation began.

Motivation for Rehabilitation

The Behavioral Regulation in Exercise Questionnaire-2 (BREQ-2) is a validated 19-item scale based on self-determination theory, used to assess behavioral regulation in exercise [30]. In the BREQ-2, questions related to why a patient engages in physical activity and exercises are asked. Their responses were measured on a five-point Likert-type scale ranging from 1 (not true) to 5 (very true). This scale has five subscales that measure five types of exercise regulation: amotivation, as well as external, introjected, identified, and intrinsic regulations. There are no specific cut-off values or standardized scores for the BREQ-2. The scores for each subscale were compared to determine the dominant motivational adjustment style. The BREQ-2 scores were measured at four time points: baseline and at the 1st, 5th, and 10th VR interventions.

Mood States

The Profile of Mood States Second Edition (POMS-2) is a reliable and validated 32-item questionnaire used to monitor mood changes and psychological stress [31]. This scale comprises subjective feelings in six different mood states: Tension, Depression, Anger, Fatigue, Confusion, and Vigor. A five-point Likert scale, ranging from 0 (not at all) to 4 (extremely), was used. The total score calculated for each mood state was converted into a standardized score. A higher score indicated more intense emotions, and a standardized score between 40 and 60 indicated a healthy state. The POMS-2 score was measured at four time points: baseline, 1st, 5th, and 10th VR interventions.

Catastrophic Thoughts of Pain

The Pain Catastrophizing Scale (PCS) is used to reliably assess the extent of magnification, rumination, and helplessness in response to episodes [32]. The PCS has 13 items, each scored on a five-point Likert scale from 0 to 4 (0 = not at all; 4 = all the time). The PCS is a total composite score (range, 0-52), with a total score of >30 representing a clinically significant level of pain catastrophizing. PCS scores were measured at four time points: baseline, 1st, 5th, and 10th VR interventions.

Quality of Life (QoL)

The 12-Item Short-Form Health Survey (SF-12) is a validated health-related QoL scale [33]. The SF-12 consists of 12 questions that are used to measure eight health concepts: physical functioning, physical role, bodily pain, general health, vitality, social functioning, emotional role, and mental health. Standardized scores, ranging from 0 to 100, were calculated for the eight subscales, with a standard value of 50. Higher scores indicate better health. The SF-12 score was measured at three time points: baseline, 5th, and 10th VR interventions.

Statistical analyses

Descriptive statistics were calculated for all participants. Statistical analysis was not performed because the sample size was insufficient to ensure adequate reliability. Continuous variables are summarized as means and standard deviations (SDs). Categorical variables are summarized as medians and interquartile ranges, or frequencies and percentages, as appropriate. R version 4.4.2 (R Foundation for Statistical Computing, Vienna, Austria) was used as the statistical software for the calculation.

## Results

Notably, we were unable to recruit 40 cases as our sample size owing to the COVID-19 pandemic, which restricted access to patients; hence, only six patients with chronic musculoskeletal pain were enrolled in the study. All participants were female (100%), with a mean age of 76.50 (SD = 8.60) years. The causes of chronic pain were musculoskeletal disorders and disabilities, including fracture (n = 1), hemiplegia after stroke (n = 2), ossification of the posterior longitudinal ligament (n = 1), osteoarthritis (n = 1), and spondylosis (n = 1). The sites of pain were the hips (n = 1), knees (n = 1), lower back (n = 1), neck (n = 1), and shoulders (n = 2) (Table 1).

**Table 1:** Participant characteristics SD, Standard deviation; BI, Barthel index

Characteristic	(n = 6)
Age (years), mean (SD)	76.50 (8.60)
Female sex, n (%)	6 (100)
BI, mean (SD)	78.33 (21.13)
Diseases, n (%)	
Fracture	1 (16.66)
Hemiplegia after stroke	2 (33.33)
Ossification of the posterior longitudinal ligament	1 (16.66)
Osteoarthritis	1 (16.66)
Spondylosis	1 (16.66)
Site of pain, n (%)	
Hip	1 (16.66)
Knee	1 (16.66)
Low back	1 (16.66)
Neck	1 (16.66)
Shoulder	2 (33.33)

All participants completed the VR home-visit rehabilitation program. The mean duration of VR usage was 857.53 (SD = 209.79) seconds. No adverse effects or dropouts were observed in this study (Table 2).

**Table 2:** VR implementation metrics SD, Standard deviation; VR, Virtual reality

VR implementation metrics	(n = 6)
Duration using VR (s), mean (SD)	857.53 (209.79)
Dropouts, n (%)	0 (0)
Adverse effects, n (%)	
Pressure pain	0 (0)
Muscle strain	0 (0)
Nausea	0 (0)
Dizziness	0 (0)
Headache	0 (0)
Others	0 (0)

**Figure 3:**
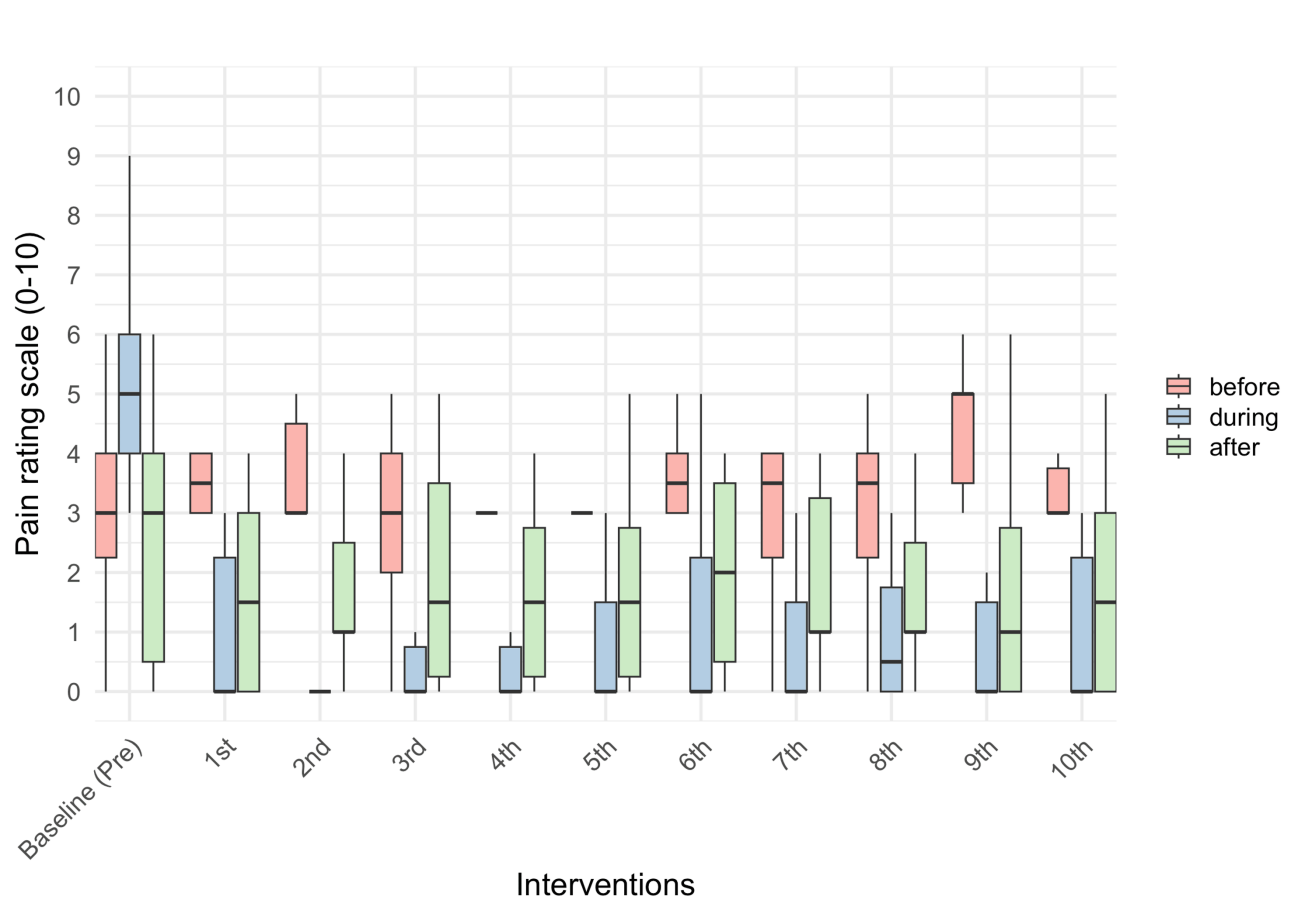
Degree of pain in home-visit rehabilitation Pain was measured at each home visit rehabilitation session and also at three time points during the rehabilitation session (before, during, and after the session). The median of the "during" value at the second rehabilitation is 0. The interquartile ranges of the "during" value at the second rehabilitation and the "before" values at the fourth and fifth rehabilitations are 0. Hence, those values were not visible. Pre, Pre-intervention

The pain was measured at three time points during the home-visit rehabilitation sessions (before, during, and after rehabilitation). The mean (or median) pain score for “before” at the baseline was 3.0; however, the scores varied between 3.0 and 5.0 during the 10 interventions. The “during” pain score at the baseline was 5.0, and the scores decreased between 0.0 and 0.5 during the 10 interventions. The “after” pain score at the baseline was 3.0, and the scores decreased between 1.0 and 2.0 during the 10 interventions (Table 3; Figure 3).

**Table 3:** Degree of pain in the home-visit rehabilitation Values are expressed as median (interquartile range). NRS, Numerical rating scale; Pre, Pre-intervention; VR, Virtual reality

Pain measured at every home-visit rehabilitation (NRS: 0-10) (n = 6)											
Time-points of measurement	VR interventions										
	Baseline (Pre)	1st	2nd	3rd	4th	5th	6th	7th	8th	9th	10th
Before rehabilitation	3.0 (1.75)	3.5 (1.0)	3.0 (1.5)	3.0 (2.0)	3.0 (0.0)	3.0 (0.0)	3.5 (1.0)	3.5 (1.75)	3.5 (1.75)	5.0 (1.5)	3.0 (0.75)
During rehabilitation	5.0 (2.0)	0.0 (2.25)	0.0 (0.0)	0.0 (0.75)	0.0 (0.75)	0.0 (1.5)	0.0 (2.25)	0.0 (1.5)	0.5 (1.75)	0.0 (1.5)	0.0 (2.25)
After rehabilitation	3.0 (3.5)	1.5 (3.0)	1.0 (1.5)	1.5 (3.25)	1.5 (2.5)	1.5 (2.5)	2.0 (3.0)	1.0 (2.25)	1.0 (1.5)	1.0 (2.75)	1.5 (3.0)

The HRV data collection was incomplete because some data points for HR, HF, and LF/HF were not collected. There were missing values of 12.5% at baseline, 33.3% at the 7th, 9th, and 10th interventions, and 16.6% at the 8th intervention; however, there were no missing values at other time points. The missing values were not supplemented. Hence, the mean and SD were calculated based on the available data. HR was 69.05 bpm (SD = 7.26) at baseline, and 64.5 bpm (SD = 9.47) to 71.76 bpm (SD = 6.48) during the intervention period. HF was 74.28 ms² (SD = 95.32) at baseline, and 33.60 ms² (SD = 25.35) to 87.12 ms² (SD = 77.12) during the intervention period. The LF/HF ratio was 2.68 (SD = 1.51) at baseline and 1.45 (SD = 0.77) to 4.28 (SD = 2.98) during the intervention period (Table 4).

**Table 4:** HRV in the home-visit rehabilitation procedures Values are expressed as mean (SD). Some data points for HR, HF, and LF/HF were not collected. Missing values were 12.5% at baseline, 33.3% at the 7th, 9th, and 10th interventions, and 16.6% at the 8th intervention; however, there were no missing values at other time points. bpm, Beat per minutes; SD, Standard deviation; VR, Virtual reality; HRV, Heart rate variability; HR, Heart rate; HF, High frequency power; LF, Low frequency power; Pre, Pre-intervention

HRV measured at every home-visit rehabilitation (n = 6)											
HRV parameters	VR interventions										
	Baseline (Pre)	1st	2nd	3rd	4th	5th	6th	7th	8th	9th	10th
HR (bpm)	69.05 (7.26)	64.5 (9.47)	67.0 (7.33)	66.9 (7.87)	66.2 (6.57)	67.6 (8.67)	66.0 (8.15)	69.78 (5.31)	71.26 (4.92)	70.72 (5.62)	71.76 (6.48)
HF (ms^2 ^)	74.28 (95.32)	84.90 (56.52)	74.61 (92.98)	63.94 (38.00)	87.12 (77.12)	33.60 (25.35)	47.07 (21.26)	69.33 (82.75)	56.22 (58.14)	49.85 (35.49)	36.32 (23.70)
LF/HF ratio	2.68 (1.51)	1.45 (0.77)	1.95 (1.67)	1.60 (0.78)	2.78 (1.97)	4.28 (2.98)	4.04 (2.64)	3.55 (2.70)	2.57 (2.52)	2.96 (2.69)	2.94 (1.26)

The motivations for home-visit rehabilitation were also studied. At all time points (baseline, 1st intervention, 5th intervention, and 10th intervention), intrinsic regulation was 13.5-14.5, and identified regulation was 13.0-14.0. On the other hand, introjected regulation was 0.0-0.5, external regulation was 0.0-1.0, and amotivation was 0.0-2.0 (Table 5).

**Table 5:** Motivation for the home-visit rehabilitation Values are expressed as median (interquartile range). Pre, Pre-intervention; VR, Virtual reality

Behavioral Regulation in Exercise Questionnaire-2 (BREQ-2) (n = 6)					
Time-points of measurment	Subscales				
	Intrinsic regulation	Identified regulation	Introjected regulation	External regulation	Amotivation
Baseline (Pre)	14.5 (4.0)	14.0 (2.75)	0.5 (1.75)	0.5 (2.5)	1.5 (3.75)
1st VR intervention	14.0 (0.75)	14.0 (0.75)	0.0 (0.0)	0.0 (0.0)	0.0 (2.25)
5th VR intervention	14.5 (4.0)	13.5 (2.5)	0.0 (0.75)	0.0 (0.75)	2.0 (1.5)
10th VR intervention	13.5 (5.25)	13.0 (5.0)	0.0 (3.0)	1.0 (3.5)	2.0 (3.5)

Participants’ mood states were stable. The standardized scores on all six POMS-2 subscales were within the standard range of 40-60 points throughout the baseline, 1st, 5th, and 10th interventions (Table 6).

**Table 6:** Mood state Values are expressed as median (interquartile range). Pre, Pre-intervention; VR, Virtual reality

Profile of Mood States Second Edition (POMS-2) standardized scores (n = 6)						
Time-points of measurement	Subscales					
	Tension	Depression	Anger	Vigor	Fatigue	Confusion
Baseline (Pre)	49 (9.25)	53 (4.5)	42.5 (10.5)	44.5 (13.25)	48 (5.5)	45 (8.25)
1st VR intervention	48 (5.0)	52 (5.0)	42 (4.25)	52 (12.5)	46 (7.0)	45 (14.25)
5th VR intervention	43 (7.25)	46 (10.25)	41 (3.75)	45.5 (11.5)	42 (4.25)	42.5 (13.25)
10th VR intervention	43 (8.75)	47 (11.25)	42 (4.25)	42 (10.0)	46 (6.25)	44 (17.75)

The PCS scores were analyzed, and the total PCS score was 28.5 at baseline, 37.0 at the first intervention, 27.0 at the fifth intervention, and 25.0 at the 10th intervention. The score at the first intervention was above the cutoff (>30) (Table 7).

**Table 7:** Catastrophic thoughts of pain Values are expressed as median (interquartile range). Pre, Pre-intervention; VR, Virtual reality

Pain Catastrophizing Scale (PCS) (n = 6)				
Time-points of measurement	Subscales			Total score
	Rumination	Magnification	Helplessness	
Baseline (Pre)	13.0 (2.25)	6.0 (3.75)	9.0 (5.25)	28.5 (10.5)
1st VR intervention	15.5 (6.25)	6.5 (3.0)	14.5 (6.5)	37.0 (15.5)
5th VR intervention	10.0 (6.25)	6.0 (4.5)	14.0 (6.5)	27.0 (17.0)
10th VR intervention	10.5 (9.75)	4.5 (4.0)	10.5 (8.25)	25.0 (12.5)

Regarding the QoL, the mental health score was above the standard value of 50. However, the scores for the other seven subscales were below the standard values at most time points. Physical functioning gradually decreased from 33.8 points at baseline to 17.2 points at the end of the intervention. In contrast, social functioning gradually increased from 31.7 points at baseline to 52.25 points at the end of the intervention (Table 8).

**Table 8:** Quality of life Values are expressed as median (interquartile range). Pre, Pre-intervention; VR, Virtual reality

12-Item Short-Form Health Survey (SF-12) standardized score (n = 6)								
Time-points of measurment	Subdomains							
	Physical functioning	Role physical	Bodily pain	General health	Vitality	Social functioning	Role emotional	Mental health
Baseline (Pre)	33.8 (8.32)	28.6 (11.2)	29.95 (11.1)	36.3 (0.0)	46.1 (22.57)	31.7 (25.42)	35.2 (16.27)	51.3 (10.15)
5th VR intervention	22.7 (8.25)	37.05 (18.3)	35.5 (8.32)	36.3 (11.32)	50.6 (6.75)	36.8 (15.3)	40.6 (4.05)	57.1 (10.15)
10th VR intervention	17.2 (19.32)	34.2 (16.87)	29.95 (11.1)	43.85 (15.1)	41.6 (13.5)	52.05 (17.75)	37.9 (9.45)	54.2 (4.35)

## Discussion

In this study, we investigated the feasibility and safety of applying VR to home-visit rehabilitation for patients with chronic musculoskeletal pain. All participants had diseases or disorders that caused chronic musculoskeletal pain. The participants were older adults, with an average age of 76.5 years. Despite their age, they all completed 10 VR-based home rehabilitation sessions without dropping out or experiencing any adverse effects. Thus, the VR-based intervention used in this study was feasible and safe. This feasibility may partly stem from the fact that the burden on the participants was low because the VR content was designed to be friendly and familiar. The VR content did not include rapid scene changes, so patients could avoid frequently moving their gaze to keep up with the changes, and the usage time was short enough (approximately 14 minutes) to prevent adverse effects [25,26].

If the NRS score was ≥4, pain was considered moderate to severe [34]. In this study, at baseline, the pain during rehabilitation was moderate or high; nonetheless, it was mild before and after the rehabilitation. A pain-alleviating intervention is considered effective when the NRS score improves by ≥2 points [35]. In this study, pain during rehabilitation decreased by ≥4.5 points from baseline (5.0) after the home-visit rehabilitation using VR began. Thus, our VR-assisted home-based rehabilitation program was effective in alleviating pain. In addition, the level of pain experienced after rehabilitation ranged from 1.0 to 1.5 for most of the VR intervention period and decreased to <0.5 of the baseline levels of 3.0. The baseline level of pain experienced was not very high; nevertheless, the resulting decrease may support the idea that pain was alleviated using VR-assisted home-based rehabilitation. This is consistent with the results of previous studies showing that VR intervention reduces acute and chronic pain during and after VR [36]. However, pain levels did not differ before rehabilitation, suggesting that the VR-mediated alleviation of pain was transient and did not persist long enough to reduce the “before rehabilitation” pain score in the next session. The pain experienced by the participants before rehabilitation persisted even though they were in a resting state. The intervention used in this study was not efficient enough in alleviating the persistent pain encountered when not viewing the VR. Hence, verification of the long-term effects is necessary.

We performed HRV analyses at every home-visit rehabilitation session to assess the balance between sympathetic and parasympathetic nervous system activities. The HR remained stable at approximately 70 bpm throughout the intervention period. In contrast, HF and LF/HF showed large fluctuations during the intervention period; hence, no clear trends were observed. However, focusing on the HF and LF/HF values at baseline and the first intervention point, a shift towards the dominance of the parasympathetic nervous system was observed, as indicated by the increase in HF and the decrease in LF/HF. Reportedly, changes in HRV are associated with the stress-reducing effects of VR, such as pain relief [24,37]. The lack of a clear trend may be due to factors such as the large degree of variation in individual differences and the extremely small sample size used in this study. Therefore, further research is required in this area.

Intrinsic and identified regulations were the predominant motivational factors for home-visit rehabilitation from baseline to the end of the intervention. Intrinsic regulation is the motivation that arises from enjoying or being satisfied with the exercise itself, whereas identified regulation refers to the process by which individuals recognize the value or importance of exercise and align it with their personal goals and values [30]. Notably, it has been reported that motivation is promoted through interactions with virtual spaces in rehabilitation using VR, such as gamification [38]. The VR content used in the present study did not involve such interactions. This may partly explain why our VR-assisted home-based rehabilitation program did not affect motivational factors. However, the VR content of rehabilitation programs can be optimized to promote motivational factors.

The participants' mood states, assessed using the POMS-2, were good. Previous studies have reported that many patients with chronic pain are depressed, which is related to the exacerbation and persistence of chronic pain [34]; however, the participants in this study did not fall into that category. This might be because the pain experienced by the participants in this study was not particularly severe, and they were able to achieve sufficient pain relief during rehabilitation.

The PCS scores of the participants were below the cut-off value at baseline, which was clinically insignificant. The score rose above the cut-off for the first intervention and fell below the cut-off for the 5th and 10th interventions. During the first intervention, pain scores (NRS) showed a decreasing trend during and after rehabilitation, whereas other psychological measures remained unchanged. As the PCS is closely related to psychological conditions, such as pain and depression [39], the rise in PCS at the first intervention may be due to the possibility that the initial intervention made patients more aware of their pain and perception. This may temporarily lead to a feeling of greater pain. However, because the PCS is highly sensitive to the efficacy of pain-relieving treatment [39], the efficacy of VR-assisted home rehabilitation needs to be further investigated.

The participants' QoL was below the standard value for most of the subscales, which is consistent with previous research showing that the QoL of patients with chronic pain was low [40]. However, their mental health status remained within the normal range, aligning with the finding that there was no clear decline in mental indicators, such as mood states, motivation for rehabilitation, and catastrophic thoughts of pain, which represents a typical characteristic of the participants. Physical functioning gradually decreased from baseline to the end of the VR intervention, while social functioning gradually increased over the same period. The changes in the physical conditions and activity levels of the participants, as well as changes in their social relationships and roles, could have influenced their physical and social functioning. However, the sample and data sizes used in that study were too small to validate this hypothesis. Future studies should adopt a larger sample size and expand on the data sets.

Limitations

This study had some limitations. First, the participants were not randomly assigned to the study, which may have introduced selection bias. Second, the study was conducted with a small sample size and included only female participants, possibly limiting the generalizability of the findings. Finally, we used a single-group pre-post comparison design, which made it difficult to determine whether the observed changes were caused by the intervention, the passage of time, or other factors.

## Conclusions

In this study, we investigated the feasibility and safety of VR-applied home-visit rehabilitation in patients with chronic musculoskeletal pain and evaluated the effects of VR interventions. The absence of adverse effects and dropouts among participants suggests that the rehabilitation is feasible and safe. Furthermore, pain during VR-based home-visit rehabilitation decreased in all participants. However, owing to the small sample size of the present study, these effects should be interpreted with caution. Future research should consider increasing the sample size and including a control group to further validate the effectiveness of VR-applied home-visit rehabilitation.
